# Antifilarial Activity of the Methanolic Extract of *Indigofera tinctoria* (Fabaceae) on Bovine Parasites (*Onchocerca ochengi*)

**DOI:** 10.1155/2022/7828551

**Published:** 2022-10-08

**Authors:** Enock Enock Rakwa, Benoît Bargui Koubala, Bertrand Ndou Mando, Mathieu Djongra, Francis Nveikoueing, Dieudonné Ndjonka

**Affiliations:** ^1^Department of Biological Sciences, University of Maroua, P.O. Box 814, Maroua, Cameroon; ^2^Department of Chemistry, University of Maroua, P.O. Box 814, Maroua, Cameroon; ^3^Department of Biological Sciences, University of Ngaoundere, P.O. Box 454, Ngaoundere, Cameroon

## Abstract

Onchocerciasis is a major public health problem caused by *Onchocerca volvulus* parasite and transmitted to humans via black flies (*simulium)* bites. The control of onchocerciasis relies much on the use of the chemical drug ivermectin, which is only effective against microfilariae and has led to drug resistance. This study was carried out to assess the *in vitro* antifilarial activity of methanolic extract of *Indigofera tinctoria* and its most active fractions on adult male *O. ochengi* worm, the closest model to *O. volvulus*, after 48 h and 72 h of treatment. Worms' viability was determined biochemically by MTT/formazan colorimetry assay. The promising plant extract's acute and subacute oral toxicity were evaluated on both mice and rats. The result revealed a highest antifilarial activity of the methanolic extract (LC_50_ = 12.28 *μ*g/mL) compared to ivermectin (LC_50_ = 26.50 *μ*g/mL) after 72 h of treatment. Out of the eight (08), chromatographic fractions screened, only three (03) fractions (C, F, and G) revealed the highest anti*-Onchocerca* activity after 72 h of treatment. An oral administration of the plant extract at a single dose of 2000 mg/kg did not produce any toxicity in mice. After repeated daily administration of methanolic extract of *I. tinctoria* (250 mg/kg, 500 mg/kg, and 1000 mg/kg) for 28 days, no significant changes in body weight, biochemical, and haematological parameters was observed. Histopathological examination of organs did not reveal any sign of alteration. The phytochemical analysis of the methanolic extract of *I. tinctoria* revealed the presence of various phenolic compounds. Therefore, this study demonstrated the potential antifilarial activity of *Indigofera tinctoria* and offered an alternative to treating onchocerciasis. Moreover, further studies could be developed in promising new antifilarial sources of the isolated compound and *in vivo* antifilarial activity of *Indigofera tinctoria* in the animal model needs to be studied.

## 1. Introduction

Onchocerciasis, or river blindness, is a parasitic disease caused by *Onchocerca volvulus* transmitted to humans via a black fly of *Simulium* genus bites. It is one of the seventeen neglected tropical diseases of major public health concerns [1, 2]. It causes severe visual impairment, including permanent blindness, skin rashes, lesions, intense itching, and depigmentation [3]. The disease is endemic in Africa and currently afflicts an estimated 37 million people globally, with 99% of all cases living in sub-Saharan Africa [4]. Almost 270,000 are blind, and more than 500,000 people are visually impaired [5].

A rapid epidemiological mapping of the onchocerciasis (REMO) survey in Cameroon revealed that about 50% of the rural population was at risk. Furthermore, the burden of the disease has important socioeconomic consequences, including long-term disability, social stigmatization, and abandonment of the infested areas that lead to economic loss [6]. In Cameroon, about 110,000 km^2^ of arable land has been left uncultivated because of the disease [7].

The principal strategy to control onchocerciasis in Africa is annual community-directed treatment with ivermectin (CDTI). Unfortunately, the filaricidal effect of this drug is only limited to microfilariae [8]. Moreover, sole reliance on ivermectin is not without limitations as recent reports indicate continuous evolution of ocular onchocerciasis even after 17 years of consistent ivermectin treatment [9]. During the treatment of onchocerciasis with ivermectin in forest zones of central Africa, several adverse events, including encephalopathy and deaths, were reported in patients coinfected with *Loa loa* [10] and the reliance of onchocerciasis control on a single drug has led to *O. volvulus* exhibiting ivermectin resistance in some communities in Ghana [11]. Therefore, a need is to screen for new, safe, and effective antifilarial drugs from natural products.

About 80% of Africa's population relies on medicinal plants for their health needs [12]. Based on the ethnobotanical survey, *Indigofera tinctoria,* a medicinal plant, is traditionally used in the north region of Cameroon against parasitic diseases (gastrointestinal infection, malaria, filariosis, and infected wound). Several studies have shown that this plant species can effectively reduce the degree of parasite infestation and is a promising alternative to conventional anthelmintic [13, 14]. Since there is no scientific report on the use of this plant against *O. ochengi* parasite, it could be a new antifilarial drug.

The model organism used for the *in vitro* test was *O. ochengi*. This bovine parasite is phylogenetically the closest species to the human filarial nematode *O. volvulus* [15], mostly used as a laboratory model of onchocerciasis [7]. This study was envisaged to scientifically evaluate the antifilarial properties of *Indigofera tinctoria* plant extract against adult worm *O. ochengi.*

## 2. Material and Methods

### 2.1. Collection and Identification of Plant Material

*Indigofera tinctoria* is a medicinal plant belonging to the Fabaceae family used to treat helmintic infections [16]. *I. tinctoria* leaves were collected from Bibemi locality in the north region of Cameroon (9°15'47 north latitude and 13°53'43 east longitude) in November 2018 based on ethnobotanical survey data. A field survey revealed that the local traditional healers used *I. tinctoria* leaves to treat intestinal helminth infections, malaria, and filarial diseases. These leaves were ground and taken as a decoction or mixed with a local wine called ‘bil-bil'; the powder is also applied to the infected wound of animals. The plant was taxonomically identified by Pr. Tchobsala, a botanist in the Department of Biological Sciences, University of Maroua, Cameroon. A voucher specimen was deposited at the National Herbarium in Yaounde and assigned number 49005/HNC.

### 2.2. Preparation of Crude Extracts and Chromatographic Fractions

*Indigofera tinctoria* leaves were dried at room temperature, ground into powder, and sieved. The powdered sample was weighed and macerated for 72 hours in three different solvents (hexane, methylene chloride, and methanol) sequentially according to the increasing polarity. After the mixture was centrifuged (3000 rpm, 10 min) and filtered, the filtrate was concentrated using a rotavapor (Buchi R-210) at appropriate temperatures. The concentrates were placed in an oven (Memmert), and the temperature was set at 40°C until all the residual solvents were evaporated. The dried crude extracts were weighed and stored at 4°C. The most active solvent phase was obtained by subjecting each extracted solvent phase to *in vitro* testing [17]. The most active crude extract was used for the bioassay-guided fractionation. Forty grams (40 g) of the most active extract (methanolic extract) was then placed in a silica gel column for chromatography and elution with the following solvents: hexane/ethyl acetate (hex/EtOAc 1 : 0-0 : 1) and ethyl acetate/methanol (EtOAc/MeOH 1 : 0-3 : 7). Collected fractions were pooled based on thin layer chromatographic (TLC) profiles. Each column fraction was then subjected to *in vitro* test for antifilarial assays [18].

### 2.3. Isolation of *Onchocerca ochengi* Adult Worms

The isolation of *O. ochengi* adult worms was done by the method described by Ndjonka et al. [7]. Fresh pieces of umbilical cattle skin with palpable nodules bought from the communal slaughterhouse of Ngaoundere II, in the Adamawa region, Cameroon, were rinsed, drained, and sterilized with 70% ethanol. Nodules were carefully excised from skin pieces with a scalpel blade and submerged directly in a phosphate buffer solution in Petri dishes. Adult male worms were carefully excised from nodules and immersed in sterile phosphate buffer solution. Afterwards, male worms were observed under a binocular microscope to determine their viability. The undamaged worms were cleaned three times in phosphate buffer solution, transferred to RPMI-1640 medium supplemented with penicillin/streptomycin (100 U/100 *μ*g/mL), and rinsed twice.

### 2.4. *In Vitro* Screening Assay of *Indigofera tinctoria* on *Onchocerca ochengi* Adult Worms

The plant extract stock and reference stock solutions were prepared. 100 mg of the crude extract was weighed and dissolved in 1 mL of distilled water in a sterile 50 mL tube (100 mg/mL). While, ivermectin stock solution was prepared by adding 10 mg of ivermectin powder to 1 mL of distilled water (10 mg/mL). All the mixtures were homogenized by stirring and kept at 4°C for further use.

Based on the protocol of Borsboom et al. [19], adult worms were incubated with different concentrations of the plant extracts ranging from 7.8 to 125 *μ*g/mL in RPMI-1640 supplemented with penicillin/streptomycin (100 U/*μ*g/mL). Ivermectin (Ivm) was used as a positive control, and DMSO was diluted in RPMI (≤2%) as a negative control. Six worms per concentration were incubated using 96-well microplates (01 worm per well of 100 *μ*L of plant products). All assays were repeated three times, and the result obtained was the mean values at each concentration after 48 h and 72 h. Lethal concentration 50 (LC_50_) was determined using SPSS 16.0 software. Crude extracts were classified after a primary screening according to the scored mortality rate as active on the worms (100%), moderately active (50-75%), and inactive (less than 50%) compared to the negative control.

### 2.5. Biochemical Assessment of Adult worm's Viability

The MTT/formazan colorimetric assay was performed to assess adult worms' viability [20]. MTT is a pale yellow compound reduced to a dark blue product formazan by mitochondrial enzymes of living cells [21]. Worms were placed under a sterile condition in a 24-well plate (six worms per well for each concentration) containing 500 *μ*L/well of 0.5 mg/mL MTT in RPMI 1640 and then incubated for 30 minutes at 37°C in the dark. After incubation, worms were removed and observed under the binocular microscope. Dead worms did not reduce MTT to formazan but took the yellow color of MTT.

### 2.6. Phytochemical Analysis

The three plant extracts were subjected to phytochemical analysis to highlight potential antifilarial secondary metabolites. The qualitative testing was done by standard staining methods of Harbone [22], Trease and Evans [23], and Sofowara [24] for polyphenols, flavonoids, tannins, alkaloids, triterpenes, sterols, and saponins identification.

### 2.7. Acute Oral Toxicity Test

An acute oral toxicity study of the methanolic extract of *Indigofera tinctoria* leaves (IT_meth_) was carried out on female mice according to guidelines 423 of the Organization for Economic Co-operation and Development (OECD) [25]. Nulliparous and nonpregnant Swiss albino mice aged 08 to 12 weeks, weighing about 20-25 g, obtained from the LANAVET (National Veterinary Laboratory of Garoua, Cameroon), were acclimatised for two (02) weeks. The animals had access to standard rodent food and water *ad labitum*. Six (06) female mice were divided into two groups (the 01 treated group and the 01 control group) of three individuals each. Before administering the plant extract, animals have fasted overnight with free access to water. A single dose of 2000 mg/kg of the methanolic extract of *Indigofera tinctoria* leaves was administered orally by gavage. The untreated group received only distilled water. Animals were observed (every 30 minutes) for their behaviour, skin changes, convulsions, diarrhoea, sleep, coma, and mortality for the first four hours, then over 14 days.

### 2.8. Subacute Oral Toxicity Test

A subacute oral toxicity test was conducted following OECD guideline 407 [26]. Twenty-four Wistar rats of both sexes (08-12-week old and weighing 125-190 g) were randomised into four groups of 06 rats each (03 males and 03 females). The methanolic extract of *I. tinctoria* leaves was administered orally to 03 groups of rats in increasing doses of 250, 500, and 1000 mg/kg of body weight. The control group received distilled water only. During 28 days, animals were treated daily and observed for clinical signs and symptoms; behaviour pattern and body weight was recorded every 02 days.

At the end of treatment, food access was restricted for 24 h and animals were anesthetized by an intraperitoneal injection of ketamine (50 mg/kg) before euthanasia. The blood sample was collected in EDTA tubes for haematological analysis, and non-EDTA tubes were used to collect and centrifuge blood at 3000 rpm for 10 minutes. The serum was kept in microtubes at 4°C for biochemical analysis. Some vital organs (kidney, liver, lung, and heart) were removed, rinsed with saline solution (0.9%), and preserved in neutral buffered formalin (10%) for histological analysis. The relative organs weight was calculated according to the following formula:

(1) $$\begin{array}{c} \text{Pr}=\frac{\text{Po}}{\text{Pa}}\text{x}100, \end{array}$$

where Po is the initial organ weight (g); Pr is the relative organ weight (g/100 g); and Pa is the weight of the rat on sacrifice day (g).

### 2.9. Haematological Analysis

After blood collection from cardiac puncture into EDTA containing tubes, haematological parameters were evaluated using an automatic haematological analyser (Nihon Kohden). These parameters included red blood cells (RBC), white blood cells (WBC), haematocrit (HCT), haemoglobin (HB), platelets, lymphocytes, and monocytes.

### 2.10. Biochemical Parameters Analysis

Various biochemical parameters were determined in serum using an automated analyser (RAL Clima MC-15). These parameters include total protein, albumin, creatinine, alanine aminotransferase (ALT), aspartate aminotransferase (AST), and urea. Protocols used in this analysis were as per the manufacturer's indication on kits.

### 2.11. Histopathological Examination of Organs

Histopathological examination of organs (liver, kidney, heart, and lung) of rats from each treated and untreated group was carried out. Tissues were fixed in 10% formaldehyde; then, organs were sequentially dehydrated. Tissues were embedded in paraffin blocks of 5 *μ*m sections made and colored by haematoxylin/eosin stains before under the optical microscope.

### 2.12. Statistical Analysis

Data were expressed as the mean value ± standard error of the mean (SEM) and analysed by one-way variance analysis (ANOVA) followed by turkey's multiple comparison test. Values were considered statistically significant at *p* < 0.05.

## 3. Results and Discussion

### 3.1. Extraction Yield

Table 1 presents the extraction yield of *Indigofera tinctoria* leaves extract with different solvents. Results showed that yields varied based on solvents used. The methanolic extract exhibited the highest yield (15.73%) compared to the methylene chloride extract (2%) and hexane extract (2.4%). The result showed a significant affinity of plant compounds to the most polar solvent (methanol).

In contradiction with Cho-Ngwa et al. [27] findings on *Homalium africanum*, the extraction yield obtained in the present work with the nonpolar extracts was low. This difference in extraction yield might be due to part, structure, and plant species as well as its chemical composition, the polarity of solvents used for extraction, method, and procedure of plant extraction [28].

### 3.2. Activity of *Indigofera tinctoria* Leaves Crude Extracts on Adult Worms

**Figure 1:**
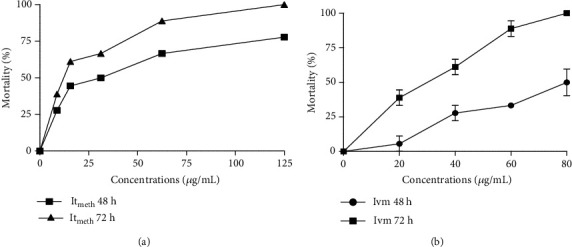
Lethal effect of the methanolic extract (a) and Ivermectin (b) concentrations on *Onchocerca ochengi* after 48 h and 72 h of incubation. It_meth_: Methanolic extract of *Indiofera tinctoria*; Ivm: ivermectin.

The methanolic extract was the most potent from the preliminary screening of the three crude extracts of *I. tinctoria*. Data shows that the methanolic extract contains polar compounds that may be the anti-*Onchocercal* agent. Hexane and methylene chloride extracts were inactive and excluded for dose-response effect assays. Both methanolic extract and ivermectin activities were time-dependent and concentration-dependent (Figures 1(a) and 1(b), respectively). This is in agreement with of Gogoi and Yadav [29], who showed the nematocidal efficacy of the methanolic extract of *Caesalpinia bonducella*. This explains the toxicity of *I. tinctoria* against adult male worms. The lowest concentration (7.8 *μ*g/mL) of the methanolic extract of *I. tinctoria* induced 22.77% and 38.88% of *O. ochengi* mortalities after 48 h and 72 h, respectively. At its highest concentrations (125 *μ*g/mL), the methanolic extract of *I. tinctoria* resulted in 77.77% and 100% of parasite mortalities after 48 h and 72 h, respectively. Previous studies have shown that some Cameroonian medicinal plants like *Anacardium occidentale, Leophira lanceolate, Adansonia digitata, Acacia nilotica, Vernonia tonoreana*, and *Cucurbita pepo ovifera* also possess anti-*Onchocercal* properties [30–32]. Thus, plants are known to demonstrate good anti-*Onchocercal* activities as chemical products.

Results presented in Table 2 displayed the highest activity of *I. tinctoria* (LC_50_ = 12.28 *μ*g/mL) compared to ivermectin (LC_50_ = 26.50 *μ*g/mL) after 72 h. There was a significant difference between the LC_50_ values of the crude extract and Ivm after 72 h. The plant showed a better *in vitro* efficacy against *O. ochengi* than Ivm. *I. tinctoria* could contain more combined actives molecules responsible for the anti-*Onchocercal* effect. This is in agreement with reports of Megnigueu et al. [33]. They also found that the ethanolic extract of *Vernonia perrottetti* displayed a very high LC_50_ compared to ivermectin and other plant extracts.

### 3.3. Activity of Chromatographic Fractions of *Indigofera tinctoria* on Adult Worm

Two hundred and seventy-one fractions of 200 mL were collected from hex/EtOAc and EtOAc/MeOH eluents. Using TLC profiles, 07 combined fractions were sequentially obtained on a chromatogram (A, B, C, D, E, F, and G) and assessed on *O. ochengi* adult male worms. According to a primary screen of fractions, only 03 chromatographic fractions (C, F, and G) were the most active against *O. ochengi.* Therefore, it may be stated that the pharmacological potential of plants is attributed to the presence of a wide array of phytochemical compounds [34, 35].

**Figure 2:**
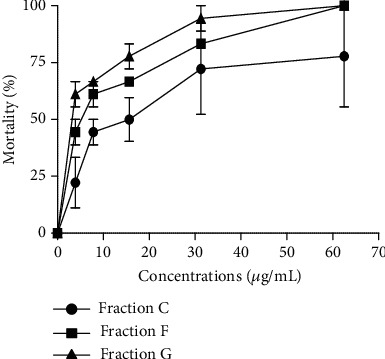
Effect of chromatographic fractions of *Indigofera tinctoria* on *Onchocerca ochengi* after 72 h of incubation.

Figure 2 shows fraction's nematocidal activity in a time and concentration-dependent manner. A similar plant product activity was demonstrated by Ndjonka et al. [7] when investigating the antinematocidal properties of *Anogeissus leiocarpus*.

The lethal concentration values of the chromatographic fractions C, F, and G are, respectively, 8.17, 3.21, and 2.83 *μ*g/mL after 72 h. The LC_50_ of these three fractions (C, F, and G) were lower than that of the crude extract and ivermectin after 72 h and consequently exhibited the highest activities on *O. ochengi* (Table 3). No difference was observed between LC_50_ values of fractions F and G (*p* < 0.001), while the crude extract's activity (LC_50_ = 12.28 *μ*g/mL) significantly differed from those of fractions F and G after 72 h. These results are similar to those of Samje et al. [21]. They evaluated the anti-*Onchocercal* activities of *Craterispermum laurinum* and *Morinda lucida* and found the higher activities of chromatographic fractions than crudes extract on *O. ochengi*. The same observation was done by Megnigueu et al. [33], revealing the potent activity of fractions against *O. ochengi* adult males with lower LC_50_ values than those of the crude extract and the ivermectin. Thus, it is likely that a fraction that kills the *O. ochengi* will also be effective against *O. volvulus* because of their high reported similarity [36, 37].

### 3.4. Phytochemical Qualities

The phytochemical screening of the plant extract revealed the presence of several secondary metabolites such as polyphenols, flavonoids, tannins, alkaloids, anthraquinone, triterpene, steroid, and saponin (Table 4).

These bioactive compounds are diverse in type and distributed in a heterogeneous manner. The presence of flavonoid, triterpens, tannins, and saponins has also been reported on *Sesbania sesban* extracts by Kumar et al. [38]. According to Bauri et al. [39], phenolic compounds, flavonoids, and tannins interfere with the energy generation mechanism and the glycoprotein of the cell surface/cuticle of parasites leading to their death. Therefore, it may be stated that the phytochemicals compound present in the methanolic extract may be responsible for the *in vitro* nematocidal effect. The qualitative and quantitative variations in phytochemical components across and within plant species are attributed to seasonal and plant maturity variation, geographical origin, genetic variation, growth stages, part of plant utilised, and postharvest drying and storage [40]. The high *in vitro* anthelmintic activity exhibited by the methanolic crude extract might be attributed to the presence of various bioactive compounds. The synergy of several compounds could contribute to the anthelmintic properties of *I. tinctoria* plant. It has been reported that phenolic compounds, including tannins and flavonoids, have been implicated in pharmacological activities such as anthelmintic [41]. It has also been proven that tannins have a high toxic effect against helmintic parasites and represent an alternative to synthetic drugs [42]. Another study stated that tannin might display its anthelmintic effect by binding to free proteins in the host animal's gastrointestinal tract or the parasite's cuticle and cause death [43]. However, this activity may also be related to the presence of saponins, considering that saponins have been reported to have anthelmintic activity [44].

### 3.5. Acute Toxicity

As the use of plant-based products increases, it is important to screen the toxicological profile of these plants to confirm the safety and efficacy of these natural sources [36]. Oral administration of *I. tinctoria* leaves extract at a single limit dose of 2000 mg/kg did not produce any signs of toxicity or mortality in all treated mice. During the observation period (02 weeks), all treated animals appeared normal and healthy without any apparent symptoms of adverse effect (posture, food and water consumption, trembling, aggressiveness, and noise sensitivity). Since no mortality was recorded and no clinical signs of toxicity with the tested doses, this suggests that the LD_50_ of It_meth_ is above 2000 mg/kg via the oral route. For the evaluation of toxicity, it was reported by Sterner and Hodge scale [45] that substances that present LD_50_ between 500 and 5000 mg/kg via the oral route may be considered practically nontoxic. Therefore, It_meth_ appears to be devoid of acute toxicity. These results are similar to those of Olurunnisola et al. [46] showing that methanolic extracts of rhizomes of *Tulbaghia violacea* were devoided of acute toxicity.

### 3.6. Subacute Toxicity

The methanolic extract of *I. tinctoria* slightly affected the rat's body weight. Males treated with 1000 mg/kg gained weight with no statistical difference from the control group. Female rats subjected to the same dose of plant extract were sensitive, so they showed weight loss with no difference compared to the control animals. Some authors showed that body weight changes are markers of adverse effects of drugs and chemicals. If the body weight loss is more than 10% of the initial body weight, it is considered statistically significant [47, 48].

### 3.7. Relative Organ Weight

Organs' weight is a highly sensitive indicator of drug toxicity [49]. The effect of methanolic extract of *I. tinctoria* on organs' weight (heart, liver, kidney, and lung) showed no significant differences (*p* > 0.05) between treated and untreated rats of both sexes (Table 5). This suggests that the oral administration of the extract (250 mg/kg, 500 mg/kg, and 1000 mg/kg) does not affect normal organ growth.

### 3.8. Haematological Studies

Haematological studies are vital indices of the pathophysiological status of animals and humans [50].  Table 6 showed the effect of It_meth_ on haematological parameters of rats after 28 days of daily treatment. It showed a significant increase in WBC, RBC, HCT, PLT, and lymphocyte (%) levels in male rats. This suggests that the It_meth_ contributed to boosting male rats' immune systems [51]. However, the It_meth_ provoked a slight decrease of WBC and haematocrit (%) in female rats; the female's immune system seems to be sensitive to this extract, the weak percentage of haematocrit in the female group can indicate an anaemia after a long treatment, but the extract did not cause any major changes (*p* > 0.05) on other haematological parameters.

### 3.9. Biochemical Studies

Transaminases such as AST and ALT are well-known indicators of liver function and are used as biomarkers to conclude the probable toxicity of drugs and xenobiotics [52]. The analysis of sera led to the compilation of data expressed in Table 7. There were no significant changes in the ALT and AST activities. Still, their activities decreased in animals of both sexes in a dose-dependent manner, suggesting that the extract did not induce any damage to the liver or kidney function of the animals. Still, the extract could rather possess hepatoprotective potential.

**Figure 3:**
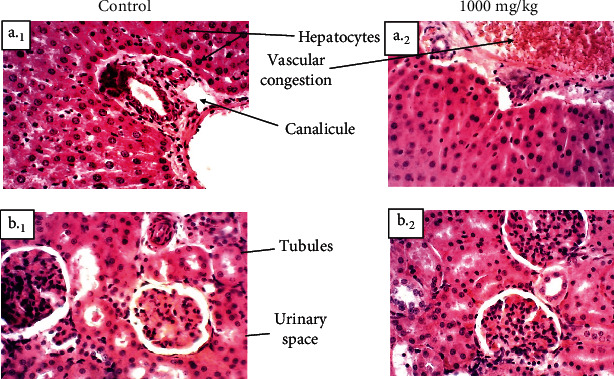
Photomicrographs of rats' liver (H&E, X200) and kidney (H&E, X200). Liver (a)_1_: control group; (a)_2_: treated group at 1000 mg/kg); kidney histology (b)_1_: control group, (b)_2_: treated group at 1000 mg/kg) of male albino Wistar rats showing relatively normal architecture. A slight liver inflammation has been noted, characterised by a slight leucocyte infiltration and vascular congestion.

An increase in the level of kidney parameters such as creatinine, urea, and uric acid in the blood is associated with reduced renal function and increased renal failure [53]. The present study shows no significant increase in the expression of kidney function markers. The normal values of these parameters indicate that repeated administration of It_meth_ did not provoke any significant renal dysfunction in animals. These findings are similar to the results of histopathological analysis, which present no kidney lesions (Figures 3(b)_1_ and 3(b)_2_).

Reductions in total protein and albumin amount are indications of diminished liver synthetic function and might be due to impaired hepatocellular function [44]. The present study showed no difference in the total protein and albumin amount in the treated group compared to the control (*p* > 0.05). This suggests that the It_meth_ did not damage organs, confirming the histopathological study that did not reveal any liver lesions (Figures 3(a)_1_ and 3(a)_2_).

## 4. Conclusion

The present study reveals that the It_meth_ possesses potential antifilarial activities against adult worms of *O. ochengi,* which is phylogenetically the closest species to the human filarial nematode *O. volvulus.* This extract offers an alternative source for developing a phytomedicine that may be used in treating onchocerciasis. The toxicity studies indicate that the consumption of the It_meth_ may be relatively nontoxic at studied doses and validates its traditional use by rural communities for treating parasitic diseases. Further investigations are ongoing on the chemical structure of active principles.

## Figures and Tables

**Table 1:** Extraction yield of *Indigofera tinctoria* leaves obtained using solvents of increasing polarity.

Mass of the powder (g)	Solvent	Mass of the extract (g)	Yield (%)
680.00	Hexane	16.32	2.40
663.68	Methylene chloride	13.25	2.00
650.43	Methanol	102.30	15.73

**Table 2:** Lethal concentrations values (LC_50_) of methanolic crude extract and ivermectin.

Time	Lethal concentrations 50 (*μ*g/mL)	
	It_meth_	Ivm
48 h	28.16 ± 2.68^b^	68.237 ± 6.28^a^
72 h	12.28 ± 3.13^b^	26.50 ± 3.48^a^

Values are mean ± SEM. Value sharing any one common superscript in a row does not differ significantly (*p* > 0.001). It_meth_: methanolic extract of *I. tinctoria*; Ivm: Ivermectin; and SEM: standard error of the mean.

**Table 3:** Lethal concentrations values (LC_50_) of methanolic crude extract and chromatographic fractions.

Incubation Times	Lethal concentrations 50 (*μ*g/mL)				
	It_meth_	FC	FF	FG	IVM
48 h	28.16 ± 2.68*^c^*	17.56 ± 5.85*^c^*	52.48 ± 7.84*^ab^*	34.19 ± 11.91*^bc^*	68.24 ± 6.28*^a^*
72 h	12.28 ± 3.13*^b^*	8.17 ± 0.87*^bc^*	3.21 ± 1.14*^c^*	2.83 ± 0.53*^c^*	26.50 ± 3.48*^a^*

Values are mean ± SEM. Value sharing any one common superscript in a row does not differ significantly (*p* > 0.001). It_meth_: methanolic extract of *I. tinctoria*; FC: fraction C; FF: fraction F; FG: fraction G; Ivm: ivermectin; and SEM: standard error of the mean.

**Table 4:** Phytochemical qualities of the crude extract of *Indigofera tinctoria* leaves.

Chemical compound	Hexane extract	Methylene chloride extract	Methanol extract
Polyphenols	—	+	++
Flavonoids	—	—	+
Tanins	—	+	++
Anthraquinon	—	—	+
Alkaloids	+	+	—
Triterpenes	+	+	+++
Sterols	+	+	+
Saponin	+	+	+

-: absent; +: present; ++: abundant; and +++: very abundant.

**Table 5:** Effect of the methanolic extract of *Indigofera tinctoria* on body organ weight after 28 days of treatment.

Treatment group		Relative organ weight			
		Heart	Liver	Kidney	Lung
Male	Control	0.760 ± 0.13^a^	7.121 ± 1.58^b^	1.463 ± 0.06^c^	2.193 ± 0.87^d^
	250 mg/kg	0.763 ± 0.11^a^	6.550 ± 1.98^b^	1.455 ± 0.40^c^	1.787 ± 0.52^d^
	500 mg/kg	0.657 ± 0.04^a^	6.804 ± 0.23^b^	1.387 ± 0.09^c^	2.034 ± 0.16^d^
	1000 mg/kg	0.746 ± 0.02^a^	7.616 ± 1.05^b^	1.712 ± 0.10^c^	2.608 ± 0.77^d^

Female	Control	0.687 ± 0.12^a^	6.250 ± 0.93^b^	1.300 ± 0.23^c^	2.162 ± 0.18^d^
	250 mg/kg	0.639 ± 0.13^a^	5.283 ± 0.96^b^	1.127 ± 0.05^c^	1.822 ± 0.46^d^
	500 mg/kg	0.676 ± 0.04^a^	5.372 ± 2.69^b^	1.283 ± 0.22^c^	1.750 ± 0.09^d^
	1000 mg/kg	0.575 ± 0.07^a^	5.220 ± 0.69^b^	1.076 ± 0.04^c^	1.542 ± 0.07^d^

Values are mean ± standard deviation of three replicates (*n* = 3). In the same column, values followed by different superscript letters are different (*p* < 0.05).

**Table 6:** Effect of the methanolic extract of *Indigofera tinctoria* on haematological parameter after 28 days treatment.

Haematological parameter	Sex	Treatment group			
		Control	250 mg/kg	500 mg/kg	1000 mg/kg
WBC count (x10^3^/*μ*L)	Males (*n* = 3)	7.88 ± 0.90^a^	11.25 ± 2.75^a^	12.26 ± 5.20^a^	12.06 ± 5.48^a^
	Females (*n* = 3)	14.75 ± 4.56^a^	9.26 ± 0.21^a^	10.55 ± 4.17^a^	8.42 ± 1.27^a^

RBC count (x10^6^/*μ*L)	Males (*n* = 3)	7.40 ± 1.07^a^	9.24 ± 0.76^a^	9.12 ± 0.65^a^	8.66 ± 1.04^a^
	Females (*n* = 3)	9.10 ± 0.98^a^	7.22 ± 0.01^a^	8.97 ± 0.86^a^	8.29 ± 2.01^a^

Haemoglobin (g/dL)	Males (*n* = 3)	12.73 ± 1.72^a^	15.03 ± 1.40^a^	14.89 ± 0.72^a^	14.79 ± 2.03^a^
	Females (*n* = 3)	14.71 ± 2.02^a^	12.88 ± 0.35^a^	14.41 ± 0.57^a^	13.75 ± 3.55^a^

Haematocrit (%)	Males (*n* = 3)	38.090 ± 4.79^a^	46.398 ± 6.55^a^	44.79 ± 3.96^a^	44.714 ± 6.81^a^
	Females (*n* = 3)	44.53 ± 5.43^a^	38.33 ± 0.21^a^	43.59 ± 4.95^a^	40.82 ± 9.04^a^

Platelet count (x10^3^/*μ*L)	Males (*n* = 3)	513.50 ± 20.00^a^	514.21 ± 21.37^a^	685.21 ± 18.14^a^	542.72 ± 15.10^a^
	Females (*n* = 3)	675.07 ± 12.01^a^	491.51 ± 21.82^a^	406.20 ± 28.79^a^	684.47 ± 20.50^a^

Lymphocytes (%)	Males (*n* = 3)	4.43 ± 0.65^a^	7.07 ± 1.88^a^	7.16 ± 2.84^a^	6.68 ± 3.63^a^
	Females (*n* = 3)	7.40 ± 2.05^a^	5.83 ± 0.35^a^	6.27 ± 3.46^a^	4.36 ± 1.16^a^

Monocytes (%)	Males (*n* = 3)	0.40 ± 0.10^a^	0.56 ± 0.15^a^	0.50 ± 0.26^a^	0.73 ± 0.49^a^
	Females (*n* = 3)	0.74 ± 0.06^a^	0.54 ± 0.07^a^	0.51 ± 0.28^a^	0.35 ± 0.06^a^

Granulocytes (%)	Males (*n* = 3)	3.03 ± 1.50^a^	3.62 ± 0.80^a^	4.60 ± 2.13^a^	4.64 ± 1.91^a^
	Females (*n* = 3)	6.60 ± 2.96^a^	2.88 ± 0.64^a^	3.76 ± 0.42^a^	3.71 ± 0.97^a^

Values are expressed as mean ± SEM. *n* = 3 females, *n* = 3 males. In each sex, the haematological parameters of the treated groups are compared to the control (ANOVA followed by multiple comparison test of Dunnett). The values sharing any one common superscript in the same row do not differ (*p* > 0.05). WBC: White Blood Cell Count, RBC: Red blood cell count.

**Table 7:** Effect of the methanolic extract of *Indigofera tinctoria* on biochemical parameters after 28 days of treatment.

Treatment group		Biochemical parameters					
		Urea (mg/L)	Creatine (mg/L)	AST (U/L)	ALT (U/L)	Albumin (g/L)	Total protein (g/DL)
Male	Control	3.41 ± 0.08^a^	5.23 ± 1.78^a^	250.08 ± 93.47^a^	117.34 ± 12.38^a^	36.24 ± 2.44^a^	2.77 ± 0.23^a^
	250 mg/kg	4.27 ± 1.20^a^	5.42 ± 0.98^a^	197.60 ± 34.53^a^	77.24 ± 17.24^a^	40.60 ± 9.08^a^	3.44 ± 0.21^a^
	500 mg/kg	3.26 ± 0.15^a^	4.89 ± 0.29^a^	192.00 ± 46.36^a^	104.08 ± 24.73^a^	36.86 ± 0.97^a^	3.10 ± 0.35^a^
	1000 mg/kg	3.26 ± 0.43^a^	5.85 ± 0.95^a^	299.27 ± 201.73^a^	83.33 ± 53.21^a^	39.00 ± 4.66^a^	3.33 ± 1.11^a^

Female	Control	3.14 ± 1.53^a^	6.23 ± 0.58^a^	258.31 ± 150.57^a^	83.88 ± 42.31^a^	38.88 ± 2.86^a^	2.87 ± 0.70^a^
	250 mg/kg	3.96 ± 0.46^a^	5.77 ± 0.99^a^	244.16 ± 70.13^a^	69.08 ± 10.34^a^	37.17 ± 2.9^a^	4.39 ± 0.64^a^
	500 mg/kg	3.56 ± 0.10^a^	6.31 ± 0.64^a^	154.70 ± 5.90^a^	71.59 ± 6.65^a^	36.94 ± 5.97^a^	3.25 ± 0.14^a^
	1000 mg/kg	3.46 ± 0.48^a^	4.81 ± 0.67^a^	244.65 ± 18.61^a^	104.72 ± 58.84^a^	33.22 ± 5.05^a^	4.69 ± 9.19^a^

Values are mean ± standard deviation of three replicates (*n* = 3). In each sex, biochemical parameters of the treated groups are compared to the control (ANOVA followed to multiple comparison test of Dunnett). The values followed by the same superscript letters in the same column are not different (*p* > 0.05).

## Data Availability

The experimental data used to support the findings of this study may be released upon reasonable request to corresponding author.
